# Inequities and inequalities in outdoor walking groups: a scoping review

**DOI:** 10.1186/s40985-020-00119-4

**Published:** 2020-03-13

**Authors:** Benjamin P. Rigby, Caroline J. Dodd-Reynolds, Emily J. Oliver

**Affiliations:** 1 Department of Sociology, Durham University, 32 Old Elvet, Durham, DH1 3HN UK; 2 NINE Doctoral Training Partnership, C/O Faculty of Social Sciences & Health, Durham University, Arthur Holmes Building, Durham, DH1 3LE UK; 3 Wolfson Research Institute, Durham University Queen’s Campus, University Boulevard, Thornaby, Stockton-on-Tees, TS17 6BH UK; 4 Fuse: The Centre for Translational Research in Public Health, Newcastle University, Newcastle-upon-Tyne, NE1 7RU UK; 5 Department of Sport and Exercise Sciences, Durham University, 42 Old Elvet, Durham, DH1 3HN UK

**Keywords:** Walking, Physical activity, Inequalities, Social determinants, Scoping review, Equity, Interventions

## Abstract

**Background:**

Outdoor walking groups are widely-used programmes aimed at improving physical activity and health outcomes. Despite being promoted as accessible and inclusive, emerging work highlights participation biases based on gender, age and socioeconomic status, for example. To explicate the impact of outdoor walking groups on physical activity inequities, we conducted a scoping review of published outdoor walking group literatures. Specifically, we critically examined: (a) equity integration strategies; (b) intervention reach; (c) effectiveness; and (d) potential social determinants of engagement relating to the World Health Organization’s conceptual framework.

**Methods:**

Arksey and O’Malley’s scoping review protocol was used to develop a comprehensive search strategy and identify relevant academic and grey literatures, which were screened using pre-defined inclusion and exclusion criteria. Data were organised by Cochrane PROGRESS-Plus equity characteristics and a narrative summary was presented for each thematic area.

**Findings:**

Sixty-two publications were included. Key findings were: (a) some evidence of targeted intervention trials. Large-scale national programmes were tailored to regional activity and health needs, which may contribute toward addressing inequities. However, participant demographics seldom informed reported analyses; (b) participation was more likely among white, more socioeconomically advantaged, middle-to-older aged, female and able-bodied adults; (c) positive physical and psychological outcomes were unlikely to extend along social gradients; and (d) interventions primarily addressed intermediary determinants (e.g. psychosocial barriers; material resource). Social capital (e.g. friend-making) was identified as potentially important for addressing physical activity inequalities.

**Conclusions:**

The published literature on outdoor walking groups leaves unanswered questions regarding participation inequalities, with implications for future physical activity promotion. Currently, participation in outdoor walking groups is typically more prevalent among advantaged subpopulations. We make recommendations for research and practice to address these issues, as well as aid the translation of existing knowledge into practice. We advocate increased focus on the social determinants of engagement. A more consistent approach to collecting and analysing participant socio-demographic data is required. Our findings also support recommendations that appropriate tailoring of universal programmes to community needs and embedding strategies to increase social cohesion are important in developing equitable programmes.

## Background

Regular physical activity is an effective way for people to reduce their risk of non-communicable diseases [1]. While some European countries exhibit high physical activity prevalence (e.g. 88% of adults in Poland meet guideline recommended levels), engagement is typically low (e.g. 12% of adults in Slovakia and 16% in Croatia), especially among socially, culturally and economically disadvantaged subpopulations [2]. Walking is increasingly promoted as part of strategies to increase physical activity [3, 4]. In particular, outdoor walking groups, popular in Westernised societies, have been proposed as a way to promote physical activity that may contribute to reducing participation-related inequalities [5]. They are typically short led walks (< 1 h), associated with increased physical activity [6] and improved health [7]. However, their success is not typically measured in terms of how well they work for those most likely to benefit [8]. Although walking involves low cost, skill and risk, engaging high-risk subpopulations remains challenging [5]. By understanding how groups support or inhibit participation of disadvantaged subpopulations, public health investment can prioritise schemes able to deliver greater health, social and economic benefit. Our paper addresses a gap identified in recent reviews [6, 7], by specifically exploring how equity is considered in these programmes and associated research. In particular, we consider so-called equity integration strategies, which refer to how publications consider the reach and effectiveness of programmes among different social groups or how programmes are designed to address known inequalities or inequities [9]. In this article, equity refers to attaining health outcomes for all people, through sustained effort to address inequalities, injustices and disparities in engagement with health promotion opportunities [10]. Inequality is the difference in participation among different social groups [11].

Numerous factors influence participation in outdoor walking groups. To date, most research has focused on individual and interpersonal factors. These include safety, physical functioning and feeling close to nature [12–14]. Furthermore, the social aspect of groups is strongly associated with participation [15, 16]. Hanson et al. suggested social factors may be less significant drivers of participation than health gains in deprived communities [8]; however, this study included participants whose exercise referrals were health-related. Generally, little is known about determinants of participation among different social groups, with social-structural determinants particularly under-examined.

An emerging body of literature from large-scale community programmes, such as Walking for Health (United Kingdom [UK]) and Heart Foundation Walking (Australia), is uncovering wider determinants of engagement in disadvantaged subpopulations. These programmes use strategies useful for addressing physical activity-related inequities [17, 18]. They foster social support, connect to multi-component campaigns and lobby community organisation, local and national government support. In particular, Ball et al. showed that individuals from sparsely-populated regions and lower income categories were overrepresented in larger walking groups [5]. However, this contrasted most previous evaluations, which suggested walkers are often women, white, socioeconomically advantaged and mid-to-older aged [6, 7, 19, 20]. Furthermore, it is unclear as to the relative impact of these factors (social support, lobbying and connecting to campaigns) and whether they apply more broadly across the literature-base. In particular, there is insufficient understanding of policy and environmental influences on participation. A comprehensive evaluation of determinants among different social groups is therefore warranted.

There have been some previous attempts to explore links between social, physical activity and health-related inequities [17]; however, the predominant focus on individual-level variables and biomedical research models has prevented inactivity being located within socio-structural causes. While increasingly popular socioecological models have offered a useful approach to considering social context, they are predominantly employed within an individually-orientated health behaviourist perspective of the social, which emphasises self-determination of behaviour [21]. A greater diversity of research and evidence is required to fully understand multiple levels of socio-structural influences on physical activity behaviour. To this end, Kay proposes the alternative social determinants of health framework [22] that places emphasis on socioeconomic and political contexts that perpetuate social hierarchies [21]. The framework also considers stratifying factors that *influence* social position (e.g. occupation, class and gender), as well as intermediary determinants that *reflect* social position (e.g. access to programmes and facilities). We test Kay’s premise in a novel context, allowing shared circumstances that influence outdoor walking groups to be identified and addressed across social gradients [22]. Given the need to critically reflect upon the predominant disciplinary perspectives contributing to physical activity research, and diversify the knowledge community in order to address inequalities [21], we examine and incorporate broad multidisciplinary perspectives. These include sociology, anthropology, psychology, epidemiology and geography, as well as service-derived material.

To do this, we also use the Cochrane PROGRESS-Plus framework [23]. This acronym summarises numerous factors known to influence opportunities to participate in and benefit from physical activity programmes: place of residence; race, ethnicity, culture or language; occupation; gender or sex; religion; education; socioeconomic status; social capital; plus age; disability; and sexual orientation [24]. Although beyond this paper’s scope to provide causal explanations, we are the first to test the PROGRESS-Plus and social determinants of health frameworks, in combination, to understand potential determinants of outdoor walking group engagement across different social groups.

Therefore, this scoping review aimed to amalgamate two methodological proposals to explore how equity and social disadvantage in outdoor walking groups is considered in published literatures. Specifically, we critically examined: (a) equity integration strategies; (b) intervention reach; (c) effectiveness; and (d) potential social determinants of engagement relating to the World Health Organization’s conceptual framework.

## Methods

We conducted a scoping review, guided by Arksey and O’Malley’s framework [25]. Scoping reviews do not present syntheses per se, rather a descriptive analytical overview of literature [26]. This approach is well-suited when the evidence-base is uncertain and may not be suitable for systematic review [6, 7]. In particular, as methodology was not of primary concern given limited available trials [25], we sought diverse literatures excluded from previous reviews. This facilitated examination of inequalities and wider social determinants of engagement [21]. We drew upon recent equity-focused reviews [9, 24] to refine our protocol. Here, we outline each review stage.

### Identifying research questions and relevant literature

The research team identified preliminary guiding questions. Through iterative discussion and initial appraisals of published literature, four research questions were determined: (1) How is equity being considered in outdoor walking group programmes? (2) What is the reach of outdoor walking groups among socially disadvantaged groups? (3) Is programme effectiveness evident within outdoor walking groups, across socially disadvantaged groups? (4) What are the social determinants of access to outdoor walking group engagement among socially disadvantaged groups, and to what extent are these addressed? Additional file 1 offers a schema for how these questions were operationalised [27].

Table 1 shows the inclusion and exclusion criteria that shaped a pragmatic search strategy [28]. We selected publications from 2012 onward to reflect developments in research and evaluation in outdoor walking groups, following recommendations in UK public health guidance on walking that called for increased attention to inequalities [29]. With regard to the exclusion criteria, the study of clinical rehabilitation or treatment interventions limits understanding of how to improve population health [30], and cohorts likely receive disproportionate support that influences participation and outcomes. Both indoor walking groups and Nordic walking require access to material resource (venues or equipment) [31, 32]. The latter is also associated with more vigorous physical activity levels and maintains its identity as a sport [32]. Further, a benefit of outdoor walking groups is that they allow participants to move at their own pace and enjoy their environment [33] without training the cardiovascular system.

**Table 1:** Publication eligibility criteria

Inclusion criteria	Exclusion criteria
- Published 2012 to date - Human adults (≥ 18 years)	- Published pre-2012 - Human youths and children (≤ 17 years) - Study investigated rehabilitation or treatment for injury or illness
- Interventions where participants (predominantly) walked in a defined outdoor walking group.	- Interventions where participants did not (predominantly) walk in a defined outdoor walking group - Study addressed (predominantly) lone walking - Study addressed (predominantly) indoor walking groups - Study addressed Nordic walking only - Participants walked at objectively prescribed intensities (e.g. HR_max_) - Review articles
- Paper or document published in English	- Paper or document not published in English

Search terms were derived iteratively, drawing on previous reviews [7, 9, 24]. The following electronic databases were searched: PubMed, Sport Discus, Cochrane Library, EMBASE and PsycINFO. We also searched the first 75 results on Google Scholar for recently published articles not yet archived, as well as databases: Grey Literature Report, Open Grey and Social Care Online. Additional grey literature known to the lead author were purposively sought (e.g. service evaluations). Initial searches generated copious publications on gait physiology, Parkinson’s disease and multiple sclerosis. Therefore, additional ‘NOT’ search terms were used. Additional file 2 contains our search syntaxes.

### Selecting publications

Systematic searches were conducted in summer 2017. We used EndNote X8 software (Clarivate Analytics) for data management and duplicate identification. Titles and abstracts were screened by the lead author who excluded irrelevant sources. Uncertainty at this stage did not preclude inclusion. All remaining articles were retrieved in full-text. Contrasting systematic reviews, double-screening is not always necessary nor feasible in scoping reviews [26]. Texts not retrieved by 4 August 2017 were excluded (*n* = 7). All full-texts were screened against inclusion and exclusion criteria by the lead author (see Table 1). Ten percent of included articles, selected at random, were screened by the two co-authors. Discrepancies were discussed and consensual resolutions applied to the screening process, resulting in four additional exclusions. Reference lists of included publications were hand-searched. These and grey literature were screened in full.

### Data charting

A data extraction sheet, adapted from [9, 24], was developed and used for all included studies and piloted on five documents [9, 27]. A revised form was developed to extract data from service evaluations and non-intervention studies. Where multiple studies addressed the same data set, companion articles were combined.

### Collating and summarising findings

This process had two phases. First, we compiled a descriptive, primarily numerical summary of publication characteristics. Second, we produced a narrative output reporting against each research question. Sources were coded, using NVivo 10 software (QSR International), initially according to which PROGRESS-Plus factors they considered. See Additional file 3 for measures of each factor included in this review. Where necessary, as authors, we discussed and resolved any uncertainty as to how these were reported in included publications. Within each factor-related node, sources were coded further according to evident equity integration strategies, reach and evidence of effectiveness. Finally, sources were coded according to the social determinants of health framework to identify potential determinants of outdoor walking group engagement. We identified gaps in the literature and summarised key findings.

## Findings

**Fig. 1:**
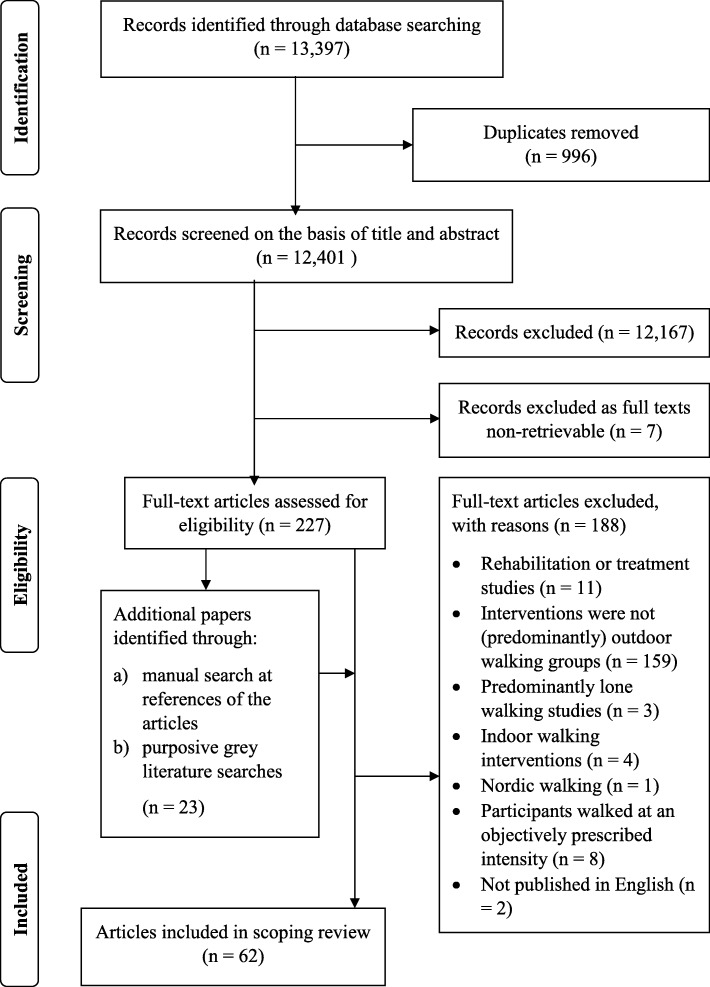
Flow chart demonstrating the search strategy conducted, and the publications that were selected, at each stage of the review process

Figure 1 details screening and selection processes from which 41 studies across 62 published documents were included in our review. Summary characteristics are provided in Additional file 4.

Forty-nine of 62 included publications were peer-reviewed, a further 12 were service evaluation reports from specific programmes, and one was a doctoral thesis. Table 2 shows research designs. Quantitative designs were cross-sectional (*n* = 7) and longitudinal (*n* = 2); five used secondary data and four generated primary data. Five studies were randomised controlled trials (RCTs). Two mixed-methods studies contained cluster-RCT and RCT components respectively. Multidisciplinary authorship produced 38 articles. The nature of remaining authorship teams were unknown (*n* = 4) or originated from eight distinct disciplines. Thirteen had sport and exercise science authors. Fifteen articles had authors directly involved in intervention design or delivery, of which seven were peer-reviewed. Works emanated from seven countries: UK (*n* = 30), USA (*n* = 20), Australia (*n* = 8). Spain, South Korea, Belgium and India (all, *n* = 1).

**Table 2:** Research designs of included studies (*n* = 41)

	n	%
Mixed methods	10	24.4
Cross-sectional	7	17.1
Qualitative	6	14.6
RCT	5	12.2
Pre-experimental	4	9.8
Longitudinal	2	4.9
Case study	2	4.9
Ethnography	2	4.9
Quasi-experimental	2	4.9
Nested RCT	1	2.4

### Equity integration strategies

With one exception [34], articles included at least one strategy (see Table 3). Fifty-five publications reported participant information on at least one PROGRESS-Plus characteristic. Age (*n* = 53) and gender and/or sex (*n* = 34) were reported most.

**Table 3:** Charting of included articles based on integration of equity strategies

Author, date and study reference [#]	Inclusion of population characteristics that relate to equity across relevant Progress-Plus factors											Further strategies	
	Place of residence (13, 21%)	Race, ethnicity, culture, or language (30, 48.4%)	Occupation (16, 25.8%)	Gender or sex (34, 54.8%)	Religion (0, 0%)	Education (23, 37.1%)	Socioeconomic status (23, 37.1%)	Social capital (22, 35.5%)	Age (53, 85.5%)	Disability (11, 17.7%)	Sexual orientation (0, 0%)	Prospective intention to focus on equity-factor, stating rationale (33, 53.2%)	Evident strategy to address socio-structural causes of inequity (0, 0%)
Adams et al. 2015, [35]			**•**				**•**	**•**	**•**			**•**	
Anderson-Lewis et al. 2012, [36]												**•**	
Baker et al. 2015, [37]		**•**		**•**		**•**	**•**		**•**			**•**	
Bang et al. 2017, [38]				**•**		**•**			**•**				
Bowen et al. 2014, [39]		**•**	**•**	**•**		**•**			**•**	**•**		**•**	
Capalb et al. 2014, [40]		**•**		**•**		**•**		**•**	**•**			**•**	
Cronin 2016, [41]	**•**	**•**		**•**			**•**		**•**	**•**			
Coulon et al. 2012, [42]			**•**	**•**		**•**	**•**	**•**	**•**			**•**	
De Moor et al. 2013, [19]	**•**	**•**		**•**				**•**	**•**	**•**			
Dodd et al. 2014, [43]							**•**		**•**				
Doughty, 2013, [44]									**•**				
Forthofer et al. 2016, [45]		**•**		**•**		**•**	**•**		**•**			**•**	
France et al. 2016, [46]	**•**	**•**		**•**		**•**	**•**		**•**				
Gilinsky, 2014, [47]			**•**					**•**	**•**			**•**	
Grant 2015, [48]	**•**												
Grant et al. 2017a, [15]	**•**							**•**	**•**	**•**			
Grant et al. 2017b, [49]	**•**							**•**	**•**	**•**			
Gusi et al. 2015, [50]				**•**		**•**		**•**	**•**			**•**	
Hanson and Jones 2015, [51]												**•**	
Hanson et al. 2016a, [52]												**•**	
Hanson et al. 2016b, [8]				**•**					**•**			**•**	
Heart Foundation 2012, [20]		**•**	**•**	**•**				**•**	**•**				
Heart Foundation 2016, [53]	**•**	**•**	**•**				**•**	**•**	**•**				
Izumi et al., 2015, [33]	**•**	**•**		**•**		**•**	**•**		**•**			**•**	
Jacobsen 2013, [54]												**•**	
Kassavou et al. 2014, [55]			**•**						**•**				
Kassavou et al. 2015a, [56]													
Kassavou et al. 2015b, [968]									**•**				
Keller et al. 2014, [57]		**•**	**•**						**•**			**•**	
Kinnafick et al. 2014, [58]			**•**	**•**					**•**				
Kokolakakis et al. 2015, [59]	**•**	**•**		**•**			**•**		**•**	**•**			
Kwarteng et al. 2017, [60]		**•**		**•**		**•**	**•**		**•**			**•**	
Lee et al. 2016, [61]			**•**					**•**	**•**			**•**	
Lord and Bush 2012, [62]			**•**					**•**	**•**	**•**		**•**	
Marselle et al. 2013a, [63]	**•**	**•**		**•**		**•**	**•**	**•**	**•**				
Marselle et al. 2013b, [33]	**•**	**•**		**•**		**•**	**•**	**•**	**•**				
Marselle et al. 2015, [64]									**•**				
Marselle et al. 2016, [65]									**•**				
Matthews et al. 2012, [66]								**•**	**•**				
Pelssers et al. 2013, [67]				**•**		**•**		**•**	**•**			**•**	
Phan et al. 2013, [68]												**•**	
Philips et al. 2012, [14]	**•**	**•**		**•**					**•**				
Raine et al. 2017, [34]													
Schoffman et al. 2015, [69]		**•**		**•**		**•**		**•**	**•**			**•**	
Schofield 2012, [70]												**•**	
Schulz et al. 2015, [71]		**•**		**•**		**•**	**•**		**•**			**•**	
Schulz et al. 2017, [72]		**•**		**•**		**•**	**•**		**•**			**•**	
Scott and Cronin 2016, [73]	**•**	**•**					**•**		**•**	**•**			
Siceloff et al. 2014, [74]			**•**	**•**		**•**	**•**	**•**	**•**			**•**	
South et al. 2013, [75]		**•**							**•**	**•**			
South et al. 2017, [76]		**•**							**•**	**•**			
Subitha et al. 2013, [77]			**•**	**•**		**•**	**•**		**•**			**•**	
Thøgersen-Ntoumani et al. 2014a, [78]		**•**		**•**					**•**			**•**	
Thøgersen-Ntoumani et al. 2014b, [79]		**•**		**•**					**•**			**•**	
Thøgersen-Ntoumani et al. 2015, [80]		**•**		**•**					**•**			**•**	
Van Wormer et al. 2012, [81]		**•**		**•**		**•**		**•**	**•**				
Vega-Lopez et al. 2015, [82]		**•**	**•**			**•**	**•**	**•**	**•**			**•**	
Wilcox et al. 2014, [83]		**•**		**•**		**•**			**•**	**•**			
Wilson 2015a, [84]		**•**		**•**			**•**		**•**				
Wilson 2015b, [85]		**•**		**•**			**•**	**•**	**•**			**•**	
Wilson et al. 2013, [86]			**•**	**•**		**•**	**•**	**•**	**•**			**•**	
Wilson et al. 2015, [87]			**•**	**•**		**•**	**•**	**•**	**•**			**•**	

Intervention studies (*n* = 14) tended to state a prospective intention to focus walking group provision towards particular social groups based on at least one PROGRESS-Plus factor, including race and ethnicity [35, 42, 57, 60, 71, 72, 74, 82, 86–88]; place of residence [39, 60, 71, 72, 77, 82]; gender [43, 47, 57, 88]; age [50, 67]; occupation [78–80]; and education [38]. In total, 33 articles included in this review aimed to examine factors associated with physical activity participation among pre-determined population subgroups. Meanwhile, just two intervention studies evaluated universal programmes [37, 54].

Contrastingly, evidence indicates that large-scale programmes may be accessible to the general population. For example, in Australia, Heart Foundation Walking is tailored and resourced according to regional physical activity and health needs [70, 84]. Selective funding and community partnerships are used to support individual rural or indigenous communities [53]. However, evidence from the UK is less clear. While trends from the Walking for Health programme suggest that provision may relate to the percentage of older people, or people living with long-term illness or disability, resident in some local authorities [41, 51], there are concerns that health inequalities may be widened where programmes are not available to, or targeted at, those in greatest need [51].

### Reach

Identified publications provided reasonable assessment of outdoor walking group reach. Generally, schemes appeared popular. For example, Walking for Health has approximately 70,000 participants across the UK [73], and 5045 adults were exposed to walking groups across the 14 identified intervention studies. When we examined reach across PROGRESS-Plus characteristics, however, walkers were predominantly women [37, 45, 46, 50, 53, 56, 59, 75, 76, 78, 82, 89, 90], white [43, 46, 75, 76, 89], socioeconomically advantaged [38, 46, 71, 72, 75, 77] and mid-to-older aged [15, 20, 40, 46, 49, 53, 55, 59, 75, 76, 90]. Overall, our assessment corroborated previous findings [6, 7, 19, 20], and contrasted with Ball et al.’s recent conclusions [5]. The Australian scheme’s tailored community support appears to be a significant factor in this.

While not mutually exclusive, each PROGRESS-Plus characteristic is relevant in itself. We found the social distribution of outdoor walking group engagement was comparable to that of total physical activity [18, 91, 92], with the exception of fewer younger adults and men. It is worth noting, however, that these programmes do seem successful at engaging women and older people, who ordinarily are underrepresented in physical activity programmes [93]. Physical activity needs to be enjoyable and purposeful, men and younger people do not necessarily find the sociable nature of walking groups to be so [15].

#### Gender or sex

Available data indicate an average of 79% of participants in universal programmes were women [14, 20, 41, 46, 53, 89]. One walking bus programme recruited no men [37]. The highest proportion of men (48.7%) was in a rural community-based programme in India [77]. Women were often represented disproportionately compared to population averages. For example, recent Walking for Health data indicated 70% female walkers [41], 15% higher than the general UK population [57]. Most men in this programme were already active before joining groups [59]. However, one study suggested men were more likely to participate as age increased [62].

#### Age

It was only possible to ascertain a grand mean age (46 years) from ten of the 14 interventions studies [35, 37, 38, 42, 43, 46, 49, 57, 58, 60, 61, 71, 72, 74, 78–80, 82, 86–88]. This figure was somewhat distorted by studies targeting older [50, 67] and younger adults [41, 47, 57, 61, 77]. Eighty-eight percent of Walking for Health participants were 55 years or older, and increasing age was the single biggest predictor of participation to 75 years in larger-scale programmes [73]. Seventy-five percent of Heart Foundation Walking participants were over 60 years [84, 85]. Other studies supported these trends [40, 81].

#### Race, ethnicity, culture or language

Alluding to the appeal of such programmes, several studies demonstrated that Hispanic and non-Latino Black subpopulations do participate in outdoor walking groups [35, 39, 42, 45, 57, 60, 69, 71, 72, 74, 79, 82, 83, 86–88]. However, all seven were based in the USA, and five specifically targeted minority ethnic groups. Large-scale programmes differed considerably. Approximately 3% of Walking for Health participants were from minority ethnic backgrounds, compared to 15% of the UK population. Similarly, 2% of Heart Foundation walkers were indigenous, and 83.5% spoke English as a first language. Two further studies described walkers as typically white [40, 81].

#### Socioeconomic status

One intervention reported income and had 50% of participants classified as more advantaged (income > $50,000 US per annum) [78]. Similarly, Walking for Health reports suggest participants were ordinarily more affluent [16, 19, 33, 41, 46, 52]. Contrastingly, Heart Foundation Walking had a disproportionately high representation of participants (22%) classified as the most disadvantaged (income < $25,000 AUS) compared to the national average (13%) [53, 84, 85].

#### Disability

One intervention in social housing developments reported that 66% of participants’ self-rated health, a strong predictor of mortality [94], as good or better [39]. Various analyses from Walking for Health indicated inequalities in participants’ health and disability statuses. Approximately 12% reported disability, despite 35% of these walks promoting themselves towards those with disabilities [41, 49, 51, 59], and 19% of working age and 45% of pension age adults in the UK having registered disabilities [16, 68]. One study indicated their provision may be higher in areas of increased disability, but not significantly [51].

### Evidence of effectiveness

Literature showed that outdoor walking groups resulted in increased physical activity (according to both objective and subjective measures) and numerous health benefits, with negligible negative outcomes. These benefits were both physical and psychological, and included increased physical fitness [50]; decreased hypertension and positive changes in blood lipoproteins [37, 60, 71, 82]; decreased breathlessness [52]; increased self-efficacy and relaxation [77]; and reductions in depression [38] and anxiety [50]. Thus, our findings largely support previous reviews [6, 7]. Here, however, we focus on differentiated outcomes. Given the evident social distribution of participation, it is likely that the interventions inadvertently exacerbated health inequities in society more generally.

The potential effect of walking groups on reducing physical activity inequalities was rarely evaluated despite the collection of demographic information. Only seven publications presented differential analyses, of which six reported differential outcomes between social groups, according to place of residence [72], occupation [77, 81], gender or sex [42, 59, 81], education [77], social capital [33] and age [77]. Primarily, these differences related to opportunities to increase physical activity levels through engagement with programmes. They are described here.

Trail users in a programme targeting African Americans were predominantly men (79%) but they participated in less total moderate-vigorous physical activity than women [42]. After a 24-week maintenance period, following an 8-week program, areas with a higher concentration of non-Latino Black residents and higher levels of poverty saw reduced participation [72]. Walking for Health evaluations indicated men were slightly more likely to adhere to walking than women (15% vs. 13% attending at least half of possible weeks over 18 months) [59], as well as being married accounting for 2.3% of variance in mental wellbeing outcomes among participants [33]. Secondary analysis of the Health Works Trial data found that being male was the strongest predictor of non-participation, although as a workplace scheme this finding may differ from leisure-based groups [81]. Finally, in the aforementioned India-based study [77], men and those 20–26 years old (compared to 40 or above) were more likely to comply with programme recommendations. Similar compliance differences were evident by level of education, whereby graduates (88%) complied the most and dropped out the least (2%). Those who had never attended school were least likely to comply and most likely to drop out. Furthermore, non-workers were six times, and semi-skilled workers were two times, more likely to comply than manual labourers.

### Potential determinants

#### Socioeconomic and political context

While we identified potential determinants of outdoor walking group participation across all dimensions of the social determinants of health framework, only six publications considered socioeconomic and political context [41, 50, 68–70, 88]. A number of reported strategies reflected known physical activity determinants [95, 96]. For example, land-use policies and national investment may enhance walkability, particularly for people with disabilities or long-term health conditions [37, 68, 70], while cultural saliency of interventions is important among ethnic minority groups [88]. One study linked walking groups to primary care services in Spain, enabling free participation for older adults [50]. It has since been shown there is insufficient evidence to determine equity in primary care-based physical activity interventions however [24]. A case study presented an interesting finding of a steering group between politicians and walkers [68]. This provided an opportunity for groups to articulate their interests and is important for reducing health inequalities [97]. Primarily, however, governance was restricted to initiatives aimed at altering social position.

#### Social position

Social position was considered in two ways. First, through targeted interventions [35, 37, 38, 42, 43, 47, 50, 57, 60, 67, 71, 72, 74, 77–80, 82, 86–88]. Second, through studies focussing on community empowerment [36, 42]. This latter strategy seemed influential in supporting participation amongst disadvantaged subpopulations by race and ethnicity, as well as by socioeconomic status. Strong partnerships fostered between members of walking groups, local organisations and status-holding institutions (e.g. faith groups or law-enforcement) were important for participant recruitment and operationalisation of local assets to support programmes [18, 40, 42, 49, 52, 55–57, 68, 70, 74, 86–88, 90]. Furthermore, it appears beneficial to have walk leaders who come from disadvantaged backgrounds. Training individuals, who then become role models, helps elevate social capital among individuals and their subpopulations [5, 42, 49, 52, 55–57, 60, 71, 72, 74, 82, 86–88, 90].

#### Intermediary

Publications most commonly considered potential intermediary determinants, including access to programmes and numerous characteristics from PROGRESS-Plus. Identified intermediary determinants are summarised here.

##### Psychosocial factors:

Certain psychosocial factors made walking groups less appealing to people from deprived areas, older adults and women. Fear of crime, dogs, stigma or negative health outcomes were all cited as barriers among these groups [39, 42, 57, 60, 71, 72, 74, 82, 86–88]. The provision of safe, low-intensity physical activities were important for older adults and in deprived areas [39, 40].

##### Time factors:

The structured and scheduled nature of many outdoor walking groups is an important consideration for subgroups by occupation, education and gender. Time of day and walk duration were commonly cited as a barrier among the employed [39, 55, 56, 58, 78–80, 90]. Similarly, a cohort of South Korean students indicated that the scheduling of walks may clash with their learning timetables [38]. Nevertheless, creating structured schedules for walks may be important to allow people to plan time and space for participation. We found this to be the case for women in three studies [35, 39, 57, 88].

##### Gendered differences:

We found some differences emerging between men and women. Men who participated in schemes did so primarily as an opportunity to share experiences with other men, in the absence of women’s company [62]. As men become older, this tendency is perhaps diminished in favour of strong women role models [62]. Contrastingly, women preferred the social elements of walking groups [42, 44] and mothers responded well to additional material support (e.g. prams or childcare) [43, 57, 88].

##### Social cohesion:

With one exception [56], we found that the capacity for outdoor walking groups to build social cohesion and social capital among participants is perhaps their most important property for addressing physical activity inequalities. Although social cohesion is intermediary, it cuts across the framework, linking higher and lower-order determinants, therefore repositioning individuals in hierarchies and consequently altering access to health resources [22]. Cohesive processes including role-modelling [69], friend-making [34] and political governance [68] were evident across PROGRESS-Plus characteristics.

##### Social support:

Specifically, in low socioeconomic status subpopulations, family commitments can be a barrier to participation [8, 13]. Therefore, companionship and family support are critical enabling factors. As such, Walking for Health and Heart Foundation Walking often attracted families without need to explicitly target them [19, 44, 70]. One study suggested that having a busy social life was negatively associated with participation [42], whereas a second found marriage to be positively associated [65]. It seems therefore that existing levels of social capital influence individuals’ likelihood of participation. Outdoor walking groups may present an opportunity for those feeling isolated and lonely, or recently separated from a romantic relationship or marriage, to make friends [15, 48, 49, 58]. This was thought to be particularly important for older adults [15, 40, 48, 49, 53, 67, 73, 84]. Trust and reciprocity are proposed mechanisms by which social capital and cohesion increase and may influence social position [15, 22, 48].

## Discussion

This scoping review has contributed to knowledge by applying the PROGRESS-Plus and social determinants of health frameworks to examine how equity and social disadvantage are considered in outdoor walking group literature. Our findings clearly show publications insufficiently and inconsistently examine these factors.

Notwithstanding the broad search strategy, the final number of included studies was small. Nevertheless, we found a diverse range of literatures from across the evidence hierarchy, including qualitative and ethnographic studies (e.g. [15, 44, 48, 49, 62]). These allowed participants’ experiences to be contextually grounded [98] and articulate how conditions constrained or enabled their physical activity. ‘Lay’ views are an important factor in considering equity in physical activity across subpopulations [21]. Equity in outdoor walking groups may be better considered by assembling these views, alongside multi-disciplinary academic perspectives, whereby deliverers are engaged the research process and can therefore initiate programme improvements [99, 100].

Our review also questions the basis on which many outdoor walking groups are developed. Community-based programmes are increasingly prevalent and are commonly underpinned by socioecological models [101]. However, there remains debate as to the extent these promote reflection beyond individually-orientated behavioural theories, and capture the complex social-structural influences on physical activity engagement [21]. The utility of these programmes and the associated support offered in engaging disadvantaged groups remains equivocal [18]. As such, equity needs to be an integral consideration of future research and practice in this field.

However, equity integration in current publications is superficial. While studies collected participant information across PROGRESS-Plus factors, notably age and gender and sex, this seldom informed analyses and has contributed little to our understanding of how outdoor walking groups may benefit particular social groups. We question therefore the extent to which this approach represents a genuine equity integration strategy as proposed [9].

The second most common strategy was to target programmes to particular subpopulations. This presents a complex dilemma for programme commissioners and practitioners, who need to decide how best to resource and tailor provision within local communities. On the one hand, evidence suggests that successfully implemented physical activity programmes, targeted towards the needs of particular subgroups, may reduce inequalities in participation [102]. However, health programmes will seldom impact on health inequalities in the longer-term, if not appropriately targeted across the entire gradient [103]. Our findings echo concerns already raised around preferential use of targeted outdoor walking groups by those in dominant or advantaged social positions [7, 104]. By design, many programmes automatically exclude much of the population [24] and if left unchecked, may reinforce inequities in such programmes that are proposed to be very accessible.

Heart Foundation Walking, and to a lesser extent Walking for Health, have demonstrated success in engaging deprived communities through community empowerment strategies [41, 85] and this needs to be more widespread in the programmes offered. We can learn from the way in which these programmes are tailored and resourced according to regional physical activity and health needs. Additional costs of tailoring programmes will likely be recouped by health-related economic gains of increased physical activity [46].

That we found intermediary determinants to be most frequently considered reflects a previous review of health promotion strategies that incorporated the social determinants of health framework [105]. While these determinants do not independently address equity, they remain important [18]. We found strategies recommended to increase physical activity in disadvantaged subpopulations [5] that addressed many known barriers to walking group engagement [13, 14]. The socially interactive aspect of these programmes is critical [7]. It is important that this is promoted and a concurrent understanding of wider structural determinants of participation is gained. Furthermore, we welcome further attempts to utilise and reflect upon similar methodological frameworks as we have done here, to elucidate such findings. This will help facilitate more inclusive outdoor walking groups.

### Limitations

Whilst this review presents a novel mapping of outdoor walking group literature, it has limitations. First, the iterative process and pragmatic search strategy inevitably led to the prioritisation of literatures. Only published studies were included, making publication bias possible, especially given the positive outcomes reported, and some relevant work may not have been included. We recommend that our findings are viewed alongside previous relevant reviews [6, 7], and older studies reporting on inequalities in walking group uptake (e.g. [106]). Second, findings were largely based on literature from the UK, Australia and the USA. This reflects the appetite for walking groups in Western societies [5, 29], in which health inequalities persist or are widening [107]. Third, the PROGRESS-Plus framework is unlikely to contain all personal characteristics affecting walking group engagement, and isolates characteristics we know intersect. Finally, the literature was reinterpreted through the descriptive analytical methodology, therefore compromising replicability. While we were unable to undertake the optional consultation phase of the scoping review process, as proposed by Arksey and O’Malley [25], the next logical step is to engage with walking group stakeholders to discuss such findings and their implications for practice and further research. It should be noted that we have been careful not to make causal claims relating to factors discussed, and our findings should not be generalised as such.

### Recommendations for research and practice

#### Equity integrations strategies

Where practicable, it is important to collect demographic information about walking group participants. However, to advance understanding of equity in these programmes, PROGRESS-Plus-related information cannot be collected as a matter of course without reason, as seems to be common here and in other fields (e.g. [108]). We recommend consistent measures be used and acted on to facilitate comparisons between social groups as routine practice in evaluation and monitoring of programmes.

Gender and sex were used synonymously across publications, despite their fundamental differences [109]. While not unique to outdoor walking group literature (indeed PROGRESS-Plus makes no distinction), it is important for researchers and practitioners to acknowledge these differences and recognise how gender’s socially constructed stratifying nature affects opportunities to access health-enhancing programmes. Furthermore, no publications reported on participants’ religion or sexual orientation. This reflects a wider lack of research examining these factors’ influence on physical activity [24]. No publications presented a strategy to reduce walking group inequalities, perhaps due to the ill-established link between physical activity and inequalities [17]. Research in these areas is required.

We identified few interventions that were designed around the principles of proportionate universalism (i.e. the resourcing and delivering of universal services at a scale and intensity proportionate to the degree of need [103]). This is perhaps indicative of cost, logistical difficulties and ongoing debate among public health academics as to how best to deliver these [18, 110]. This reinforces the need to develop and evaluate such programmes [21]. There is evidence, from Heart Foundation Walking for example, to suggest that outdoor walking groups can be made available to all, not only targeted towards disadvantaged groups, but in a way that still ensures a responsiveness to levels of presenting needs within communities.

#### Reach

There is evident need to explore walking groups’ reach among people of differing religion and sexual orientation. No studies targeted subpopulations according to disability, as such the effectiveness of this approach remains unknown. It was also difficult to ascertain the reach of walking groups by socioeconomic background due to disparate measures used. Our reach findings should be interpreted cautiously, not least due to the highly targeted nature of intervention studies, and the known recruitment biases in physical activity research more generally [92, 111]. Furthermore, inconsistent reporting, especially for education and socioeconomic status, hindered assessments. However, the general distribution of walking group participation indicates an imperative to ensure programmes are designed according to the best available evidence on addressing physical activity inequities [17, 18], while research explores ways to better engage traditionally disadvantaged subpopulations.

#### Evidence of effectiveness

No difference in health outcomes were found among different social groups participating in outdoor walking groups. The majority of PROGRESS-Plus characteristics were not associated with differences in physical activity. However, where differences were observed, it was not possible to discern which characteristics were attributable. This, and the small number of available analyses, highlights the need for further research into differentiated effects. Nevertheless, generally positive outcomes suggest that some participants in different social groups may benefit from programmes in ways not captured by existing research practices. We encourage diversity in research approaches that capture the voices of those typically excluded from physical activity opportunities.

While we did not exclude by design, the lack of RCTs was notable, given recent recommendations for such evaluations [24, 112]. Where an aggregated estimation of effect is required, we advocate this approach. Where individual studies may be insufficiently powered to undertake subgroup analyses, consistent reporting of intervention effects in subgroups across studies will allow subsequent pooling to establish differential intervention characteristics among specific subpopulations [112]. We argue that there is a role for more contextual, in-depth evaluation however, and an expanded evidence-base as presented here [21]. This will require a close relationship between programmes and the research community, drawing upon the expertise of practitioners and participants alike.

#### Potential determinants

Studies did not easily map on to the social determinants of health framework. This is perhaps due to the extent community-based interventions are designed according to socioecological models [101]. We support Kay’s calls for researchers to consider the social determinants of health framework therefore and encourage them to build on our efforts [21]. Furthermore, there is insufficient information about the socioeconomic and political context determinants of outdoor walking group engagement. These are seldom considered in programmes, a shortcoming to redress. Given walking groups’ ability to create social capital and cohesion [15, 48, 49], future programmes should harness this. Further research is required to ascertain the cohesive mechanisms and if they differ between population subgroups. Meanwhile, those promoting walking groups may wish to emphasise the benefits of social interaction and offer opportunities for walkers to become involved in delivery of programmes in their local communities.

## Conclusion

We have attempted to present an accessible overview of published literature, regarding inequity and inequality in outdoor walking groups. These programmes may improve physical and psychological health of participants. However, we suggest that the outdoor walking groups (and their benefits) may be preferentially accessed by traditionally advantaged subpopulations. We observed inequalities comparable to general trends in physical activity participation, thus corroborating concerns in recent systematic reviews. Sustained efforts to understand and reduce these gaps are necessary.

Walking group programmes primarily addressed proximal intermediary determinants and seldom considered socioeconomic and political context. These shortcomings will likely inhibit attempts to empower social groups and increase access to such community health opportunities [63]. Nevertheless, the association between these programmes and social capital may help to reorganise individuals’ social position and access to physical activity opportunities. Furthermore, we support increased tailoring of programmes to community health needs.

We advocate further equity-related research and encourage attention towards social determinants of engagement. This may help reinforce links between physical inactivity and health inequalities, while facilitating more equitable access to the positive health outcomes associated with outdoor walking groups.

## Supplementary information

**Additional file 1.** Schema of research questions operationalization.

**Additional file 2.** Search syntax for electronic database.

**Additional file 3.** Included measures of PROGRESS-Plus factors.

**Additional file 4.** Summary of characteristics of included publications.

## Data Availability

All data analysed during this study are cited in the reference list..

## Abbreviations

UK: United Kingdom
RCT: Randomised controlled trial
USA: United States of America
