# Supplementary material for: Inequities and inequalities in outdoor walking groups: a scoping review
Source: Public Health Rev. 2020 Mar 13;41:4. doi: 10.1186/s40985-020-00119-4 (PMC7071574; doi:10.1186/s40985-020-00119-4)
Supplement: Supplementary file 1 — Additional file 1. Schema of research questions operationalization. [file 40985_2020_119_MOESM1_ESM.pdf]

Supplementary file 1 Schema of research questions operationalisation

Research question	Objectives	Purpose	Operational definitions
1. How is equity being considered in outdoor walking group programmes?	Determine the extent to which equity is considered within outdoor walking group studies.<br><br>Determine the strategies and mechanisms by which equity is integrated within outdoor walking group studies.	A greater understanding of the strategies and mechanisms by which equity considerations are integrated into outdoor walking group studies, will identify gaps in the literature, as well as an understanding of how they may address PA inequities.	Equity categories based on PROGRESS-Plus:<br>• including population characteristics across all relevant social strata;<br>• prospective intention to focus on equity, providing stated rationale<br>• evident strategy to address socio-structural causes of inequity<br>Intervention types.
2. What is the reach of outdoor walking groups among socially disadvantaged groups?	Determine the social distribution of outdoor walking group participation.	A greater understanding of which social groups are engaging may help identify gaps to target additional research, and develop more equitable service delivery.	Equity categories based on PROGRESS-Plus.<br>Participation rates reported by authors.
3. Is programme effectiveness evident within outdoor walking groups, across socially disadvantaged groups?	Determine successful exemplar programmes.<br><br>Identify within these any key components or strategies for success.	A greater understanding of which interventions are effective, may lead to better-informed public health decision making and potentially inform the development of future group walking programmes.	Effectiveness includes cost, logistics and achievement of defined outcome measures:<br>• Outcomes presented by author(s)<br>• Author(s)'s suggestions for future research
4. What are the social determinants of access to outdoor walking group engagement among socially disadvantaged groups, and to what extent are these addressed?	Determine the key contextually-situated factors which may influence outdoor walking group engagement.	A greater understanding of the influences underpinning access to outdoor walking groups, will aid in establishing links between PA and health inequities, identify gaps in the literature and improve policy and practice to widen participation.	Social determinants and solutions<br>• As stated by the authors
