# Supplementary material for: Inequities and inequalities in outdoor walking groups: a scoping review
Source: Public Health Rev. 2020 Mar 13;41:4. doi: 10.1186/s40985-020-00119-4 (PMC7071574; doi:10.1186/s40985-020-00119-4)
Supplement: Supplementary file 2 — Additional file 2. Search syntax for electronic database. [file 40985_2020_119_MOESM2_ESM.pdf]

Additional file 2 Search syntax for electronic databases

Sources searched	Search strategy used, including any limits
PubMED	*(All fields)* "walk* program*" OR "walk* intervention" OR "health walk*" OR "walk* group*" OR "walk* club*" OR "lay led walk*" OR "community based walk*" OR "community walk*" OR "walk* scheme*" OR "walk* for health" OR "group physical activity" OR “walking in groups” OR “group walk*” OR “led walk” OR “group exercise”<br><br>**Filters:** humans; 01/01/2012 to date; adults: 19+ years; English language
Sport Discus	*All fields)* "walk* program*" OR "walk* intervention" OR "health walk*" OR "walk* group*" OR "walk* club*" OR "lay led walk*" OR "community based walk*" OR "community walk*" OR "walk* scheme*" OR "walk* for health" OR "group physical activity" OR “walking in groups” OR “group walk*” OR “led walk” OR “group exercise”<br><br>*(Title)* NOT “Parkinson*” NOT “multiple sclerosis” NOT “gait”<br><br>**Filters:** January 2012 to date; English language
Cochrane Library	*(All fields)* "walk* program*" OR "walk* intervention" OR "health walk*" OR "walk* group*" OR "walk* club*" OR "lay led walk*" OR "community based walk*" OR "community walk*" OR "walk* scheme*" OR "walk* for health" OR "group physical activity" OR “walking in groups” OR “group walk*” OR “led walk” OR “group exercise”<br><br>*(Title, abstract, keywords)* NOT “Parkinson*” NOT “multiple sclerosis” NOT “gait”<br><br>**Limits:** 2012 to 2017
EMBASE	*All fields)* ‘walk* program*' OR 'walk* intervention' OR 'health walk*' OR 'walk* group*' OR 'walk* club*' OR 'lay led walk*' OR 'community based walk*' OR 'community walk*' OR 'walk* scheme*' OR 'walk* for health' OR 'group physical activity' OR 'walking in groups' OR 'group walk*' OR 'led walk' OR 'group exercise'<br><br>*(Title)* NOT 'Parkinson*' OR 'multiple sclerosis' OR 'gait'<br><br>*(Abstract)* NOT 'Parkinson*' OR 'multiple sclerosis' OR 'gait'<br><br>*(Keywords)* NOT 'Parkinson*' OR 'multiple sclerosis' OR 'gait'<br><br>**Limits:** Humans; 2012 to current; English language; Adult <18 to 64 years>; Adult <65 years +; Conference abstracts/Embase
PsycINFO	"walk* program*" OR "walk* intervention" OR "health walk*" OR "walk* group*" OR "walk* club*" OR "lay led walk*" OR "community based walk*" OR "community walk*" OR "walk* scheme*" OR "walk* for health" OR "group physical activity" OR “walking in groups” OR “group walk*” OR “led walk” OR “group exercise”<br><br>*(Title, abstract, keywords)* NOT “Parkinson*” OR “multiple sclerosis” OR “gait”<br><br>**Limits**: Publication date Jan 2012 to July 2017; adulthood (18 yrs & older); human; English language
