# Supplementary material for: Inequities and inequalities in outdoor walking groups: a scoping review
Source: Public Health Rev. 2020 Mar 13;41:4. doi: 10.1186/s40985-020-00119-4 (PMC7071574; doi:10.1186/s40985-020-00119-4)
Supplement: Supplementary file 3 — Additional file 3. Included measures of PROGRESS-Plus factors. [file 40985_2020_119_MOESM3_ESM.pdf]

Additional File 3: Included measures of PROGRESS-Plus factors

Adapted from Attwood *et al.* (2016)[1], Oliver *et al.* (2008)[2] and O'Neill *et al.* (2014)[3]:

- **P**lace of residence
  Urban/rural/inner-city classifications; perceptions of local environment (e.g. the Neighbourhood Environment Walkability Scale)

- **R**ace, ethnicity, culture and language
  Racial or ethnic group classifications (White/mixed or multiple ethnicity/Asian/Black/other), mother tongue or country of origin

- **O**ccupation
  Unemployed/employed/retired; manual or non-manual work; full-time or part-time employment; graded hierarchies measuring occupational status or prestige

- **G**ender and sex
  Male or female classifications

- **R**eligion
  Self-reported religious denomination; details of belief systems or values held; religious activities engaged in or attendance at religious institutions

- **E**ducation
  Number of years in full-time education; educational attainment or qualifications achieved; institutions attended (e.g. School/FE/HE)

- **S**ocioeconomic status
  Poverty level; income (continuous level or proportion falling into income brackets), asset-based measures such as car ownership or housing tenure; receipt of state welfare or health payment assistance

- **S**ocial capital
  Perceptions of social norms surrounding trust or reciprocity; social support (e.g. marital or living status, household size, social support from friends, family or relevant others); social networks; civic participation or group membership

Plus Factors

- Age – *mean or median age of participants; age brackets*

- Disability – *measures of functioning; health status; quality of life; physical tests of functioning*

- Sexual Orientation – *e.g. hetero-/homo/bi-/trans-sexual classifications*

---

[1] Attwood S, van Sluijs E, Sutton S. Exploring equity in primary-care-based physical activity interventions using PROGRESS-plus: a systematic review and evidence synthesis. Int J Behav Nutr Phy Act. 2016;13:60
[2] Oliver S, Kavanagh J, Caird J, Lorenc T, Oliver K, Harden A. Health promotion inequalities and young people's health. A systematic review of research. London: University of London; 2008
[3] O'Neill J, Tabish H, Welch V, Pettircrew M, Pottie K, Clarke M. Applying an equity lens to interventions: using PROGRESS ensures consideration of socially stratifying factors to illuminate inequities in health. J Clincial Epidemiol. 2014;61:56–64
