# Supplementary material for: Inequities and inequalities in outdoor walking groups: a scoping review
Source: Public Health Rev. 2020 Mar 13;41:4. doi: 10.1186/s40985-020-00119-4 (PMC7071574; doi:10.1186/s40985-020-00119-4)
Supplement: Supplementary file 4 — Additional file 4. Summary of characteristics of included publications. [file 40985_2020_119_MOESM4_ESM.pdf]

Additional file 3 Summary of characteristics of included publications

Peer reviewed intervention studies

ID #	Authors and reference numbers [#]	Name	Group characteristics (all groups)	Setting	Design	Delivered mode	Duration and timing of walking group component	Physical activity assessment	Measure-ments	Delivered by
1	Adams *et al.* [35]	Not stated	African American women (n = 29). Mean age: 48.75	Religious organisation	Pre-experimental	Self-help manuals (Stanford Walking Kit)	Once per week, for 6 weeks (flexible vs. fixed timings)	IPAQ-SF[1]	Baseline<br>7 weeks	Researcher
2	Baker *et al.* [56]	Not stated	Overweight or obese adults (n = 45). Ages: 23 to 63	Community	Pre-experimental	Not stated	Weekdays, for 8 weeks	Pedometers	Baseline<br>8 weeks	Not stated
3	Bang *et al.* [55]	Not stated	Students (n = 118). Mean age: 24.3	Higher educational	Quasi-experimental	Education lectures, Self-help manuals, Reminder texts	Once per week for 1 hour, for 6 weeks	IPAQ-SF<br>Fitbits	Baseline<br>7 weeks<br>3 months	Not stated
4	Bowen *et al.* [46]	Not stated	Adults in public housing (n = 102). Ages: 18 to 76	Community	Quasi-experimental	Not stated	At least once per week, for 12 weeks	Pedometers	Baseline<br>12 weeks	Professionals[2]
5	Coulon *et al.* [36],<br>Siceloff *et al.* [42],<br>Wilson *et al.* [44, 45]	PATH[3]	Underserved African American adults (n = 434). Mean age: 51.19	Community	RCT[1]	Education events, Social marketing resources, Calendar	Six days per week (not Sundays), for 12 months	Pedometers<br>Accelerometers	Baseline<br>12 months<br>18 months<br>24 months	Lay people[4]
6	Dodd *et al.* [48]	LIMIT	Overweight or obese antenatal women (n = 582). Mean age: 29.4	Hospital	Nested RCT	One-to-one sessions, Self-help manuals, Telephone calls	3 times per week for 40 mins, for 10-20 weeks gestation to 4 months postpartum	SQUASH[1]<br>Workbooks	Baseline, 28 and 36 weeks gestation,<br>4 months postpartum	Researcher

7	Gilinsky [49]<br>Lee *et al.* [80]	MAMMiS[3]	Postnatal women (n = 65). Mean age = 33.1	Community	RCT	One-to-one sessions, Self-help manuals	Once per week for 30-50 mins, for 10 weeks	Pedometers<br>Accelerometers<br>7-day recall questionnaire	Baseline<br>3 months<br>6 months	Professionals
8	Gusi *et al.* [50]	ELAY[3]	Older adults (n = 3,214). Mean age = 68.63	Community	RCT	Not stated	3 times per week for 50 mins, for 6 months	Functional reach, chair sit-and-reach, 3-m TUG[1], bi-handgrips, 6-min walk test	Baseline<br>6 months<br>12 months	Professionals
9	Izumi *et al.* [37]<br>Kwarteng *et al.* [39]<br>Schulz *et al.* [40, 41]	WYHH[3]	Adults (n = 603). Mean age = 40.75 years.	Community	Mixed methods (including cluster RCT)	Not stated	3 times per week for 90 mins, for 32 weeks	Pedometers	Baseline<br>4 weeks<br>8 weeks<br>32 weeks	Professionals<br>Lay people
10	Jacobsen [57]	Not stated	Adults (n = 7)	Religious organisation	Pre-experimental	Self-help manuals	Twice per week, duration not stated	Pedometers	Not stated	Lay people
11	Keller *et al.* [38]<br>Vega-Lopez *et al.* [37]	Madres para la Salud	Sedentary postpartum Hispanic women (n = 44). Mean age = 28.2	Community	RCT	Group support sessions	Once per week for 30 mins, for 12 weeks	SBAS[1]<br>Pedometers<br>Accelerometers	Baseline<br>6 months<br>12 month	Professionals
12	Kinnafick *et al.* [79]<br>Thøgersen-Ntoumani *et al.* [52-54]	Step by Step	Adults (n = 75)	Workplace	Mixed methods (including RCT)	Reminder texts	3 times per week for 30 mins, for 10 weeks	Self-report diaries<br>Pedometers	Baseline to 16 weeks	Lay people

13	Pelssers *et al.* [51]	Every Step Counts	Older adults (n = 580). Mean age = 69.6	Community	RCT	Group support sessions, Self-help manuals	Once per week, for 10 weeks	GLTEQ[1] 6-min walk test	Baseline 10 weeks	Lay people
14	Subitha *et al.* [47]	Not stated	Adults (n = 458). Ages: 20 to 49	Community	Pre-experimental	One-to-one sessions, self-help manuals	4 times per week for 30 mins, for 10 weeks	IPAQ	Baseline 10 weeks	Professionals Lay people

[1]**List of measurement abbreviations:** IPAQ-SF: International Physical Activity Questionnaire Short Form; RCT: randomised controlled trial; SQUASH: Short Questionnaire to Assess Health-enhancing Physical Activity; TUG: Timed Up-and-Go test; SBAS: Stanford Brief Activity Survey; GLTEQ: Godin Leisure Time Exercise Questionnaire
[2]**Professionals =** people who have previous education and work on behaviour change interventions as part of their profession.
[3]**List of intervention name abbreviations:** PATH: Positive Action for Today's Health; ELAY: Exercise Looks After You; WYHH: Walk Your Heart to Health
[4]**Lay people** = people who received basic training to deliver the intervention, that is not part of their professional role.

Additional peer reviewed studies which considered components of outdoor walking groups

ID #	Authors and reference numbers [#]	Design	Sample characteristics	Measures or topic(s) of investigation	Description of programme(s) or intervention(s) studied	Name	Setting	Duration and timing of walking group component
15	Anderson-Lewis *et al.* [91]	Mixed methods	Research staff (n = 14)<br>Advisory board (n = 18)<br>Walking coaches (n = 29)	Community capacity	Community-based participatory, multi-component intervention, targeting African Americans. Delivered by lay people, using culturally tailored education sessions, health screening and pedometers	HUB City Steps	Community	At least twice per month for 90 mins, for 12 months.
16	Capalb *et al.* [72]	Qualitative	Walking group participants (n = 10). Ages: 60 to 85	Barriers and facilitators	Multi-component health intervention delivered by trained instructors. Included a variety of exercises	Not stated	Community	6 months
17	Doughty [92]	Ethnography	Walking group participants (n = 40) Ages: 20 to 79	Embodied social practices and therapeutic landscapes	Five different lay led walking groups within the UK countryside	Not stated	Not stated	Not stated
18	Forthofer *et al.* [64], Schoffman *et al.* [83], Wilcox *et al.* [84]	Mixed methods	Community members (n = 62)<br>Walking group participants (n = 236)<br>Walking group leaders (n = 60)	Programme features<br>Demographic characteristics<br>Outdoor recreation area use	Programme encouraged lay walking leaders to recruit members through existing social networks and organise walking groups on an *ad hoc* basis	SCOTM![1]	Community	6 months
19	Grant *et al.* [15, 73]	Ethnography	Walking group participants (n = 19)	Qualitative value of walking groups for older adults	Walking groups delivered by the researcher, as part of national UK programme, which offers a range of intensity walks which are either open access or targeted	Walking for Health	Community	Several per week for 75-90 mins, ongoing
20	Hanson and Jones [62]	Quantitative (secondary/ cross-sectional).	Not applicable	Health and socio-economic population need	Lay led walking groups, as part of national UK programme, which offers a range of intensity walks which are either open access or targeted	Walking for Health	Community	Various frequencies, 30-90 mins, ongoing

21	Hanson *et al.* [85]	Qualitative	Group stakeholders (n = 12)<br>Walking champions (n = 9)	Programme logistics	Community walking groups delivered as part of a national programme by lay people. Targeted at those from low socioeconomic backgrounds	Walking Cities	Community	3-4 times per week, for 15 months
22	Hanson *et al.* [8]	Qualitative	Walking group participants (n = 10)	Barriers and facilitators	Community walking groups delivered by a trained physical activity leader. Targeted at those from low socioeconomic backgrounds	Not stated	Community	1-2 times per week for 50 mins, for 12 weeks
23	Kassavou *et al.* [67, 68, 74]	Mixed methods	Walk leaders (n = 18)<br>Walking group participants (n =122)	Environmental influences<br>Walkers' needs and expectations<br>Self-efficacy<br>Habituation<br>Satisfaction	Lay led walking groups, as part of national UK programme, which offers a range of intensity walks which are either open access or targeted	Walking for Health	Community	Various frequencies, 30-90 mins, ongoing
24	Kokolakakis *et al.* [68]	Quantitative (secondary/ cross-sectional)	Walking group participants (n = 64,933)	Barriers and facilitators<br>Attendance patterns	Lay led walking groups, as part of national UK programme, which offers a range of intensity walks which are either open access or targeted	Walking for Health	Community	Various frequencies, 30-90 mins, ongoing
25	Lord and Bush [78]	Qualitative	Male walking group participants (n = 184)	Barriers and facilitators	Groups were established out of three pre-existing men's well-being groups	Not stated	Community	24 weeks
26	Marselle *et al.* [110]	Quantitative (secondary/ longitudinal)	Walking group participants (n = 1,516)	Well-being	Lay led walking groups, as part of national UK programme, which offers a range of intensity walks which are either open access or targeted	Walking for Health	Community	Various frequencies, 30-90 mins, ongoing
27	Marselle *et al.* [33]	Quantitative (secondary/ cross-sectional)	Walking group participants (n = 708)	Well-being	Lay led walking groups, as part of national UK programme, which offers a range of intensity walks which are either open access or targeted	Walking for Health	Community	Various frequencies, 30-90 mins, ongoing

28	Marselle *et al.* [93]<br>Marselle *et al.*, [111]	Quantitative (primary/ longitudinal)	Walking group participants (n = 127)	Well-being	Lay led walking groups, as part of national UK programme, which offers a range of intensity walks which are either open access or targeted	Walking for Health	Community	Various frequencies, 30-90 mins, ongoing
29	Matthews *et al.* [112]	Qualitative	Walking group coordinators (n = 28)	Recruitment strategies	Considered programmes delivered by three agencies across England and Scotland	Not stated	Not stated	Not stated
30	Raine *et al.* [34]	Qualitative	Walking group participants (n = 8)<br>Walking leaders (n = 6)	Barriers and facilitators	Recruited from a programme facilitated by Stepping Stones to Nature and the YMCA	Not stated	Not stated	Not stated
31	South *et al.* [71]	Mixed methods	Walking group participants (n = 77)<br>Walking group befrienders (n = 2)	Barriers and facilitators<br>Well-being	Lay led walking groups, as part of national UK programme, which offers a range of intensity walks which are either open access or targeted	Walking for Health	Community	Various frequencies, 30-90 mins, ongoing
32	Van Wormer *et al.* [82]	Quantitative (secondary/ cross-sectional)	Adults (n = 642)	Physical activity<br>Programme participation	A multi-component worksite walking and dietary intervention including clubs which were coordinated by researchers and staff. Group walks were held periodically	HealthWorks	Workplace	7 blocks of 6-8 weeks, for 24 months

[1]**List of intervention name abbreviations:** SCOTM!: Sumter County on the Move!

Non-peer reviewed publications

ID #	Authors and reference numbers [#]	Type	Purpose of study or publication	Description of programme(s) or intervention(s) studied	Name	Setting	Research design for collated data
33	Cronin [58]	Programme evaluation	To audit and provide an analysis of the programme for 2015-16	Lay led walking groups, as part of national UK programme, which offers a range of intensity walks which are either open access or targeted	Walking for Health	Community	Quantitative (primary/ cross-sectional)
34	De Moor *et al.* [19]	Programme evaluation	To audit the programme for 2012	Lay led walking groups, as part of national UK programme, which offers a range of intensity walks which are either open access or targeted	Walking for Health	Community	Quantitative (primary/ cross-sectional)
35	France *et al.* [65]	Programme evaluation	Provide evidence of the programme's impact on health to commissioners	Lay led walking groups, as part of national UK programme, which offers a range of intensity walks which are either open access or targeted	Walking for Health	Community	Mixed methods
19	Grant [94]*	Ethnographic evaluation	To provide programme agencies with participants' perceived value of their walking groups	Walking groups delivered by the researcher, as part of national UK programme, which offers a range of intensity walks which are either open access or targeted	Walking for Health	Community	Ethnography
36	Heart Foundation [20]	Programme evaluation	To provide an overview of programme achievements for 2012	Locally coordinated, lay-led walking groups, as part of a national network in Australia. Walkers can join an online community and receive incentives for completed walks. Either open access or targeted	Heart Foundation Walking	Community	Mixed methods
37	Heart Foundation [61], Wilson, [60, 81]	Programme evaluation	To provide an overview of programme achievements for 2015-16	Locally coordinated, lay-led walking groups, as part of a national network in Australia. Walkers can join an online community and receive incentives for completed walks. Either open access or targeted	Heart Foundation Walking	Community	Mixed methods

38	Phan *et al.* [87]	Policy and practice brief	To support planning and policies that promote walking, and provide policy recommendations	Led by an active group of parents, who assess and remove barriers to safe walking.	Get Moving Kern and Greenfield	Community	Case study
39	Philips *et al.* [14]	Programme evaluation	To identify if the proportion of active participants had increased	Lay led walking groups, as part of national UK programme, which offers a range of intensity walks which are either open access or targeted	Walking for Health	Community	Mixed methods
40	Schofield [59]	Programme report	To showcases programmes from rural communities	Locally coordinated, lay-led walking groups, as part of a national network in Australia. Walkers can join an online community and receive incentives for completed walks. Either open access or targeted	Heart Foundation Walking	Community	Case study
41	Scott and Cronin [63]	Programme evaluation	To provide an overview of programme delivery in 2016	Lay led walking groups, as part of national UK programme, which offers a range of intensity walks which are either open access or targeted	Walking for Health	Community	Quantitative (primary/ cross-sectional)
31	South *et al.* [70]*	Programme evaluation	To report the effectiveness of the Walking for Wellness pilot to stakeholders	Walking groups delivered by the researcher, as part of national UK programme, which offers a range of intensity walks which are either open access or targeted	Walking for Health	Community	Mixed methods

* These papers are additional non-peer reviewed reports from studies detailed under additional peer reviewed publications. As such these have no unique identifying numbers and are not considered as separate research designs in the manuscript.
